# Association of Fluid Status and Body Composition with Physical Function in Patients with Chronic Kidney Disease

**DOI:** 10.1371/journal.pone.0165400

**Published:** 2016-10-31

**Authors:** Shih-Ming Hsiao, Yi-Chun Tsai, Hui-Mei Chen, Ming-Yen Lin, Yi-Wen Chiu, Tzu-Hui Chen, Shu-Li Wang, Pei-Ni Hsiao, Lan-Fang Kung, Shang-Jyh Hwang, Mei-Feng Huang, Yi-Chun Yeh, Cheng-Sheng Chen, Mei-Chuan Kuo

**Affiliations:** 1 Department of Nursing, Kaohsiung Medical University Hospital, Kaohsiung, Taiwan; 2 Division of General Medicine, Department of Internal Medicine, Kaohsiung Medical University Hospital, Kaohsiung, Taiwan; 3 Division of Nephrology, Department of Internal Medicine, Kaohsiung Medical University Hospital, Kaohsiung, Taiwan; 4 Graduate of Medicine, Kaohsiung Medical University, Kaohsiung, Taiwan; 5 Department of Occupational Therapy, College of Health Sciences, Kaohsiung Medical University, Kaohsiung, Taiwan; 6 Faculty of Renal Care, Kaohsiung Medical University, Kaohsiung, Taiwan; 7 Institute of Population Sciences, National Health Research Institutes, Miaoli, Taiwan; 8 Department of Psychiatry, Kaohsiung Medical University Hospital, Kaohsiung, Taiwan; Hospital Universitario de la Princesa, SPAIN

## Abstract

**Background:**

Impairment of physical function and abnormal body composition are the major presentations in patients with chronic kidney disease (CKD). The aim of this study is to investigate the relationship between body composition and physical function in CKD patients.

**Methods:**

This cross-sectional study enrolled 172 of CKD stages 1–5 from February 2013 to September 2013. Handgrip strength (upper extremity muscle endurance), 30-second chair-stand test (lower extremity muscle endurance) and 2-minute step test (cardiorespiratory endurance) were used as indices of physical function. Body composition, including fluid status (extracellular water/total body water, ECW/TBW), lean tissue index (LTI), and fat tissue index (FTI), was measured using a bioimpedance spectroscopy method.

**Results:**

All patients with high ECW/TBW had lower handgrip strength and 30-second chair-stand than those with low ECW/TBW (P<0.001 and P = 0.002). CKD patients with high FTI had lower handgrip strength and 30-second chair-stand than those with low FTI (P<0.001 and P = 0.002). These patients with low LTI had lower handgrip strength than those with high LTI (P = 0.04). In multivariate analysis, high ECW/TBW was positively associated with decreased handgrip strength (β = -41.17, P = 0.03) in CKD patients. High FTI was significantly correlated with decreased times of 30-second chair-stand (β = -0.13, P = 0.01). There was no significant relationship between body composition and 2-minute step test.

**Conclusions:**

Our results show a significant association of impaired upper and lower extremity muscle endurance with high fluid status and fat tissue. Evaluation of body composition may assist in indentifying physical dysfunction earlier in CKD patients.

## Introduction

Impairment of physical function is one of the major complications in patients with chronic kidney disease (CKD). Poor physical function is associated with not only low quality of life [1] but also increased risk for all-cause mortality [2,3] in CKD patients on dialysis or not.

CKD itself is a pro-inflammatory status [4]. The reduction of energy and protein intake and metabolic acidosis also induces abnormalities of nutrition in CKD progression [5,6]. Malnutrition-inflammation complex syndrome aggravates accumulation of metabolic waste products that may disturb the balance in body composition in CKD patients [7]. Fluid overload is a common phenomenon in CKD patients, and the abnormal distribution of fluid status is significantly associated with malnutrition-inflammation [8]. Our previous reports demonstrated a strong association of fluid overload with renal progression, cardiovascular events and all-cause mortality [9,10].

In older populations, these metabolic waste products generated by malnutrition-inflammation contributes to skeletal muscular dysfunction, which has an important influence on impairment of mobility and physical performance, consequently increasing the risks for fractures, disability, hospitalization or mortality [11]. However, the relationship between body composition and physical function has not been well-explored in CKD patients not on dialysis. We hypothesized that the change in body composition is significantly associated with poor physical function in CKD patients. The aim of this study was to investigate the association of body composition, including fluid status, lean tissue mass and fat tissue mass with physical function, including upper and lower extremity muscle endurance and cardiorespiratory endurance in patients with CKD stages 1–5 not on dialysis.

## Subjects and Methods

### Study Participants

This cross-sectional study invited 172 of CKD stages 1–5 patients to participate in the study from February 2013 to September 2013 at one hospital in Southern Taiwan. All patients had been enrolled in our integrated CKD program for more than 3 months. The integrated CKD program provided multidisciplinary care plan, including physical, nursing and nutritional treatment. We monitored renal function every 3 months at least. CKD was staged according to K/DOQI definitions and the estimated glomerular filtration rate (eGFR) was calculated using the equation of the 4-variable Modification of Diet in Renal Disease (MDRD) Study [12]. We excluded participants having instability, congestive heart failure New York Heart Association (NYHA) class III~IV, obstructive lung disease, maintenance dialysis, and shunt implantation. The study protocol was approved by the Institutional Review Board of the Kaohsiung Medical University Hospital (KMUH-IRB-20120203). Informed consents were obtained in written form from patients and all clinical investigations were conducted according to the principles expressed in the Declaration of Helsinki. The patients gave consent for the publication of the clinical details.

### Assessment of physical function

This study used handgrip, 30-second chair-stand test and 2-minute step test as indices of physical function. These examinations were performed at the same time with measurement of body composition and laboratory parameters. Handgrip strength (kgf) as an indicator of upper extremity muscle endurance was measured using a handheld dynamometer (Takei scientific instruments Co., LTD, Japan). Every participant was in a seated position and two consecutive measurements were performed at intermediate forearm position with the elbow flexed at 90 degree. The mean of the maximal values for each hand was used for analysis [13]. Lower extremity muscle endurance was measured using 30-second chair-stand test [14]. The number of times raised to a full stand in 30 seconds in each participant was used in analysis. Cardiorespiratory endurance was determined using the 2-minute step test. Participants alternately raised each knee to the midway between the patella and illac crest for 2 minutes and the numbers of repetitions were recorded [15,16]. Exercise capacity was evaluated using the questionnaire to record kinds of exercise and times.

### Measurement of body composition

Body composition was determined once by a bioimpedance spectroscopy method, Body Composition Monitor (BCM, Fresenius Medical Care), at enrollment. BCM has been validated against standard reference measures for evaluation of body composition and fluid status in CKD and normal population [8,17–20]. The BCM presented body composition as a three-compartment model, consisting of lean tissue index (LTI, normohydrated lean tissue), fat tissue index (FTI, normohydrated adipose tissue), and fluid status. LTI and FTI of the whole body were determined based on the difference of impedance in each tissue [17,19]. Marcelli et al. reported 10^th^-90^th^ percentile and >90^th^ percentile of LTI and FTI relative to age and sex matched healthy population [20]. The information of fluid status, including total body water (TBW), extracellular water (ECW), and intracellular water (ICW) were determined from the measured impedance data.

### Data Collection

Demographic and clinical data of patients were obtained from interviews and medical records at enrollment. The body mass index (BMI) was calculated as the ratio of weight in kilograms divided by square of height in meters. Blood pressure was recorded as the mean of two consecutive measurements with 5-minute intervals, using one single calibrated device. Mean arterial pressure was calculated as 2/3 diastolic blood pressure plus 1/3 systolic blood pressure. Hypertension, diabetes, hyperlipidemia and gout were defined as those with a medical history through chart review. Cardiovascular disease was defined as a history of heart failure, acute or chronic ischemic heart disease, and myocardial infarction. Blood and urine samples were obtained at the same time of fluid status measurement. Patients were asked to fast for at least 12 hours before blood sample collection for the biochemistry study. The severity of proteinuria was determined using urine protein-creatinine ratio (PCR).

### Statistical Analysis

Continuous variables were expressed as mean±SD or median (25^th^, 75^th^ percentile), as appropriate, and categorical variables were expressed as percentages. Skewed distribution continuous variables were log-transformed to attain normal distribution. The significance of differences in continuous variables between groups was tested using independent t-test or the Mann-Whitney U analysis, as appropriate. The difference in the distribution of categorical variables was tested using the Chi-square test.

Physical function in CKD patients is well-known to be lower than normal individuals whose physical function varied based on age, sex, and race [21]. There was no solid evidence of cut-off values for physical function measures in CKD group till now. It needs more studies to validate the physical function in CKD group. Thus, we used linear regression to analyze the relationship between physical function and body composition in CKD patients. All the variables in Table 1 tested by univariate analysis and those variables with P-value less than 0.05, plus age, sex, BMI, eGFR, and exercise capacity were selected in multivariate stepwise linear regression analysis. Statistical analyses were conducted using SPSS 18.0 for Windows (SPSS Inc., Chicago, Illinois) and the graphs were made by GraphPad Prism 5.0 (GraphPad Software Inc., San Diego CA, USA). Statistical significance was set at a two-sided p-value of less than 0.05.

**Table 1:** The clinical characteristics of study subjects stratified by CKD stages.

	Entire Cohort (n = 172)	CKD stage1-2 (n = 44)	CKD stage 3–5 (n = 128)	P-value
Demographic variables				
Age, year	67.1±7.7	64.8±6.0	67.8±8.2	0.01
Sex (female), %	55.2	54.5	55.5	0.91
Smoke, %	27.9	15.9	32.0	0.04
Alcohol, %	16.9	6.8	20.3	0.03
Cardiovascular disease, %	14.6	6.8	17.3	0.08
Diabetes, %	36.0	6.8	46.1	<0.001
Hypertension, %	69.2	36.4	80.5	<0.001
Hyperlipidemia, %	39.0	9.1	49.2	<0.001
Gout, %	16.3	2.3	22.1	0.004
Body Mass Index, kg/m^2^	24.3±3.8	23.8±3.7	24.6±4.0	0.22
Mean arterial pressure, mmHg	96.4±12.5	91.3±13.0	98.1±12.0	0.002
Exercise capacity, %				<0.001
No exercise	4.8	0	5.8	
Low	70.1	46.2	75.2	
Middle	21.1	34.6	18.2	
High	4.1	19.2	0.8	
Body Composition				
Lean tissue Index, kg/m^2^	14.0±2.7	14.1±2.4	13.9±2.8	0.75
Fat tissue Index, kg/m^2^	9.7±4.2	9.3±3.4	9.9±4.5	0.41
Total body water, L	32.6±6.2	32.5±6.9	32.7±6.0	0.86
Intracellular water, L	17.5±3.5	17.7±3.9	17.4±3.4	0.62
Extracellular water, L	15.2±2.9	14.8±3.1	15.3±2.8	0.29
ECW/ICW	0.9±0.1	0.8±0.1	0.9±0.1	0.001
ECW/TBW, %	46.6±3.1	45.6±2.0	47.0±3.2	0.001
TBW/BW, %	52.0±6.2	52.2±5.3	51.9±6.5	0.80
Physical function				
2-minute step	105.5(92.3,116.0)	108.5(101.0,123.7)	103.5(89.5,112.0)	0.007
Handgrip strength, kg	29.2±8.5	29.3±8.5	26.4±8.5	0.04
30-second chair stand	11.2±3.1	11.9±3.7	10.9±3.2	0.10
Laboratory parameters				
Blood urea nitrogen, mg/dl	31.5(20.0,51.4)	15.5(12.8,18.6)	38.5(28.5,57.4)	<0.001
eGFR, ml/min/1.73m^2^	37.9±31.0	86.1±13.7	21.3±12.3	<0.001
Hemoglobin, g/dl	11.0±2.0	14.3±0.9	10.9±1.9	0.001
Albumin, g/dl	4.2±0.3	4.7±0.1	4.2±0.3	<0.001
Phosphate, mg/dl	4.1(3.5,4.6)	3.2(2.9,3.4)	4.2(3.6,4.6)	<0.001
Sodium, meq/L	139.1±2.5	140.3±1.7	138.7±2.7	<0.001
Potassium, meq/L	4.2±0.5	4.0±0.3	4.3±0.5	<0.001
Cholesterol, mg/dl	187.3±35.5	200.2±28.5	182.9±36.7	0.007
Urine PCR>1g/g, %	39.7	0	44.1	0.001

*Notes*: Data are expressed as number (percentage) for categorical variables and mean±SD or median (25^th^, 75^th^ percentile) for continuous variables, as appropriate.

Abbreviations: CKD, chronic kidney disease; ECW, extracellular water; ICW, intracellular water; TBW, total body water; eGFR, estimated glomerular filtration rate; urine PCR, urine protein-creatinine ratio.

## Results

### Characteristics of entire cohort

The comparison of clinical characteristics between groups based on CKD stages is shown in Table 1. The study population consisted of 172 patients with a mean age of 67.1±7.7 years and 55.2% of male. Among them, 36.0% were diabetes and 14.6% had cardiovascular disease. The mean eGFR of all participants was 37.9±31.0 ml/min/1.73m^2^, and 44 (25.6%) were CKD stage 1–2, and 128 (74.4%) were CKD stage 3–5. The mean of ECW/TBW, LTI and FTI was 46.6±3.1%, 14.0±2.7 kg/m^2^, and 9.7±4.2 kg/m^2^ respectively. CKD stage 3–5 patients had higher ECW/TBW than CKD stage 1–2. However, there was no significant difference of LTI and FTI among different CKD stages.

### Physical function of entire cohort

Of all CKD patients, the mean of handgrip strength and 30-second chair stand was 29.2±8.5 kg and 11.2±3.1 respectively. The median of 2-minute step was 105.5(92.3,116.0). CKD stage 3–5 patients had lower handgrip strength (26.4±8.5kg *v*.*s*. 29.3±8.5kg, P<0.001, Table 1) and 2-minute step (103.5 *v*.*s*. 108.5, P = 0.007) than CKD stage 1–2 patients.

### Determinants of handgrip strength in CKD

**Fig 1:**
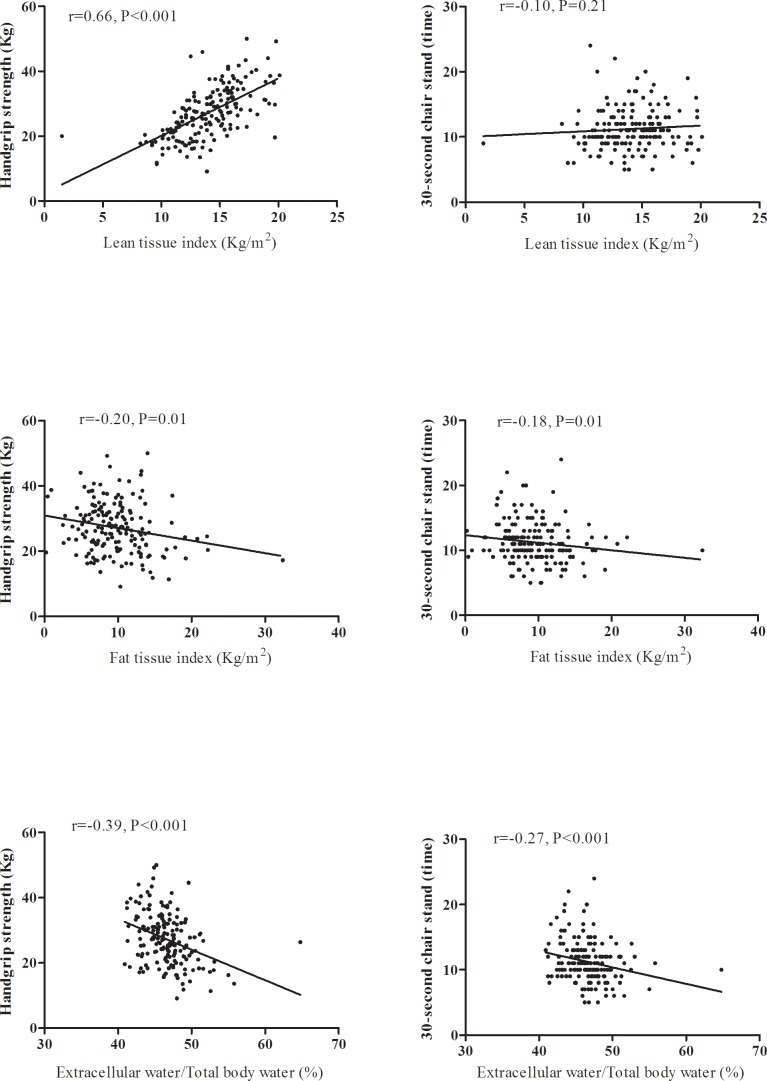
The correlation between body composition and physical function in patients with CKD stage 1–5.

Low handgrip strength was significantly correlated with high ECW/TBW (r = -0.39, P<0.001), high FTI (r = -0.20, P = 0.01), and low LTI (r = 0.66, P<0.001, Fig 1).The determinants of handgrip strength in CKD stages 1–5 patients are reported in Table 2. Handgrip strength was correlated positively with body mass index, LTI, eGFR, hemoglobin, serum albumin and potassium levels, but negatively with age, female gender, FTI, ECW/TBW, serum phosphate level, and urine PCR in univariate linear regression analysis. Further multivariate stepwise analysis showed that 1 year increase in age was related to 0.18kg decrease in handgrip strength (95% confidence index (CI): -0.31,-0.05, P = 0.006). Female was correlated with 8.30kg decrease in handgrip strength than male (95% CI: -10.61,-5.99, P<0.001). One kg/m^2^ increase in BMI was associated with 0.46kg increase in handgrip strength than male (95% CI: 0.19,0.72, P = 0.001). After adjusting age, sex, BMI, eGFR, exercise capacity, and variables of P<0.05 in univariate analysis, 1% increase in ECW/TBW was correlated with 41.17kg decrease in handgrip strength (95% CI: -79.35,-2.98, P = 0.03).

**Table 2:** The determinants of handgrip strength in patients with chronic kidney disease stage 1–5.

	Univariate		Multivariate^a^ (stepwise)		Multivariate^b^ (stepwise)	
	β(95%Cl)	P-value	β(95%Cl)	P-value	β(95%Cl)	P-value
**Clinical characteristics**						
Age, year	-0.22(-0.37,-0.07)	0.004	-0.18(-0.31,-0.05)	0.005	-0.18(-0.31,-0.05)	0.006
Sex (female)	-10.30(-12.11,-8.49)	<0.001	-9.12(-11.04,-7.20)	<0.001	-8.30(-10.61,-5.99)	<0.001
Heart disease	-0.24(-3.61,3.13)	0.88	--	--	--	--
Diabetes	-1.90(-4.35,0.55)	0.12	--	--	--	--
Hypertension	-0.16(-2.73,2.40)	0.90				
Hyperlipidemia	-1.97(-4.39,0.44)	0.10				
Gout	2.03(-1.17,5.22)	0.21	--	--	--	--
Body mass index, kg/m^2^	0.36(0.06,0.66)	0.02	0.56(0.32,0.81)	<0.001	0.46(0.19,0.72)	0.001
Mean arterial pressure, mmHg	0.04(-0.06,0.13)	0.40	--	--	--	--
Exercise capacity	3.67(2.05–5.34)	<0.001				
**Body composition**						
Lean tissue index, kg/m^2^	1.76(1.43,2.10)	<0.001	--	--	0.41(-0.05,0.87)	0.07
Fat tissue index, kg/m^2^	-0.38(-0.66,-0.11)	0.006	--	--	--	--
ECW/ICW	-32.25(-43.33,-21.17)	<0.001	-21.21(-31.56,-10.85)	<0.001	--	--
ECW/TBW	-93.13(-129.51,-56.76)	<0.001	--	--	-41.17(-79.35,-2.98)	0.03
**Laboratory data**						
Blood urea nitrogen, mg/dl	-0.05(-0.10,0.01)	0.08	--	--	--	--
eGFR, ml/min/1.73m^2^	0.04(0.01,0.18)	0.02	--	--	--	--
Fasting sugar, mg/dl	0.01(-0.03,0.04)	0.54				
Hemoglobin, g/dl	1.11(0.48,1.74)	0.001	--	--	--	--
Albumin, g/dl	4.02(0.64,7.40)	0.02	--	--	--	--
Phosphate, mg/dl	-1.60(-3.10,-0.10)	0.03	--	--	--	--
Sodium, meq/L	-0.48(-0.97,0.01)	0.06	--	--	--	--
Potassium, meq/L	2.20(-3.63,0.76)	0.003	--	--	--	--
Cholesterol, mg/dl	-0.01(-0.05,0.02)	0.48	--	--	--	--
Urine protein-creatinine ratio >1g/g	-3.13(-5.73,-0.52)	0.02	--	--	--	--

Abbreviations: eGFR, estimated glomerular filtration rate; ECW, extracellular water; ICW, intracellular water; TBW, total body water

^a^adjustment of ECW/ICW.

^b^adjustment of ECW/TBW.

We arranged subgroup analysis and found that high ECW/TBW and low LTI were significantly associated with decreased handgrip strength in males, females, patients with CKD stage 1–2, those with CKD stage 3–5, those with age of 65 years or less, and those with age above 65 years (S1 and S2 Figs). FTI was negatively correlated with handgrip strength in patients with age above 65 years and patients with CKD stages 3–5, but not in other groups (S3 Fig).

### Determinants of 30-second chair-stand in CKD

Fig 1 shows that FTI was significantly and negatively associated with the times of 30-second chair-stand in CKD stage 1–5 patients (r = -0.18, P = 0.01; r = -0.27, P<0.001 respectively). There was no significant correlation between LTI and 30-second chair-stand. In univariate linear regression analysis, the times of 30-second chair-stand were correlated positively with hemoglobin and serum albumin level, but negatively with age, gout, FTI, ECW/TBW, body mass index, and serum phosphate level (Table 3). Further multivariate stepwise analysis showed that 1 year increase in age was related to 0.1 decrease in times of 30-second chair-stand (95% CI: -0.16,-0.04, P<0.001. CKD patients with gout was related to 1.35 decrease in times of 30-second chair-stand (95% CI: -2.46,-0.23, P = 0.02) than those without gout. One g/dl increase in hemoglobin was correlated with 0.48 increase in times of 30-second chair-stand (95% CI: 0.24,0.71, P<0.001), One kg/m^2^ increase in FTI was associated with 0.13 decrease in times of 30-second chair-stand (95% CI: -0.24,-0.02, P = 0.01).

**Table 3:** The determinants of 30-second chair-stand test in patients with chronic kidney disease stage 1–5.

	Univariate		Multivariate^a^ (stepwise)		Multivariate^b^ (stepwise)	
	β(95%Cl)	P-value	β(95%Cl)	P-value	β(95%Cl)	P-value
**Clinical characteristics**						
Age, year	-0.06(-0.13,-0.00)	0.04	-0.10(-0.16,-0.04)	<0.001	-0.10(-0.16,-0.04)	<0.001
Sex (female)	-0.31(-1.27,0.66)	0.53	--	--	--	--
Heart disease,	-0.35(-1.72,1.01)	0.60	--	--	--	--
Diabetes	-0.44(-1.45,0.55)	0.38	--	--	--	--
Hypertension	-0.72(-1.75,0.31)	0.17				
Hyperlipidemia	-0.88(-1.86,0.10)	0.08				
Gout	-1.38(-2.66,0.09)	0.03	-1.35(-2.46,-0.23)	0.02	-1.35(-2.46,-0.23)	0.02
Body mass index, kg/m^2^	-0.12(-0.25,0.00)	0.05	--	--	--	--
Mean arterial pressure, mmHg	0.03(-0.01,0.06)	0.17	--	--	--	--
Exercise capacity	3.70(2.05–5.34)	<0.001				
**Body composition**						
Lean tissue index, kg/m^2^	0.09(-0.09,0.26)	0.32	--	--	--	--
Fat tissue index, kg/m^2^	-0.12(-0.23,-0.00)	0.04	-0.13(-0.24,-0.02)	0.01	-0.13(-0.24,-0.02)	0.01
ECW/ICW	-8.16(-13.00,-3.31)	0.001	--	--	--	--
ECW/TBW	-25.21(-40.75,-9.67)	0.002	--	--	--	--
**Laboratory data**						
Blood urea nitrogen, mg/dl	-0.01(-0.03,0.01)	0.19	--	--	--	--
eGFR, ml/min/1.73m^2^	0.01(-0.00,0.03)	0.11	--	--	--	--
Fasting sugar, mg/dl	-0.01(-0.02,0.01)	0.41				
Hemoglobin,g/dl	0.37(0.11,0.62))	0.005	0.48(0.24,0.71)	<0.001	0.48(0.24,0.71)	<0.001
Albumin, g/dl	1.31(0.03,2.59)	0.04	--	--	--	--
Phosphate, mg/dl	-0.59(-1.14,-0.04)	0.03	--	--	--	--
Sodium, meq/L	0.05(-0.15,0.24)	0.65	--	--	--	--
Potassium, meq/L	-0.56(-1.50,0.38)	0.24	--	--	--	--
Cholesterol, mg/dl	0.01(-0.01,0.02)	0.39	--	--	--	--
Urine protein-creatinine ratio >1g/g	-0.07(-1.06,0.93)	0.89	--	--	--	--

Abbreviations: eGFR, estimated glomerular filtration rate; ECW, extracellular water; ICW, intracellular water; TBW, total body water.

^a^adjustment of ECW/ICW.

^b^adjustment of ECW/TBW.

### Determinants of 2-minute step in CKD

In univariate linear regression analysis, the times of 2-minute step were correlated positively with eGFR, but negatively with age (Table 4). Further multivariate stepwise analysis showed that decreased times of 2-minute step were significantly associated with old age and low eGFR. There was no significant association between body composition and 2-minute step in patients with CKD stages 1–5.

**Table 4:** The determinants of 2-minute step test in patients with chronic kidney disease stage 1–5.

	Univariate		Multivariate (stepwise)	
	β(95%Cl)	P-value	β(95%Cl)	P-value
**Clinical characteristics**				
Age, year	-0.00(-0.00,0.00)	0.03	-0.00(-0.00,0.00)	0.01
Sex (male)	-0.02(-0.05,0.02)	0.32	--	--
Heart disease	0.03(-0.01,0.08)	0.15	--	--
Diabetes	-0.03(-0.06,0.01)	0.11	--	--
Hypertension	-0.02(-0.06,0.01)	0.20		
Hyperlipidemia	-0.01(-0.04,0.02)	0.55		
Gout	-0.02(-0.06,0.03)	0.46	--	--
Body mass index, kg/m^2^	-0.00(-0.01,0.00)	0.50	--	--
Mean arterial pressure, mmHg	0.00(-0.00,0.00)	0.46	--	--
Exercise capacity	0.03(0.01–0.05)	0.004	0.03(0.00,0.04)	0.03
**Body composition**				
Lean tissue index, kg/m^2^	0.00(-0.00,0.01)	0.54	--	--
Fat tissue index, kg/m^2^	-0.00(-0.01,0.00)	0.24	--	--
ECW/ICW	-0.15(-0.30,0.01)	0.06	--	--
ECW/TBW	-0.39(-0.90,0.11)	0.12	--	--
**Laboratory data**				
Blood urea nitrogen, mg/dl	-0.00(-0.00,0.00)	0.12	--	--
eGFR, ml/min/1.73m^2^	0.00(0.00,0.00)	0.003	0.00(0.00,0.00)	0.01
Fasting sugar, mg/dl	0.00(-0.00,0.00)	0.48		
Hemocrit,%	0.01(-0.00,0.01)	0.29	--	--
Albumin, g/dl	0.01(-0.04,0.05)	0.70	--	--
Phosphate, mg/dl	0.00(-0.02,0.02)	0.75	--	--
Sodium, meq/L	0.00(-0.01,0.01)	0.64	--	--
Potassium, meq/L	-0.01(-0.04,0.02)	0.41	--	--
Cholesterol, mg/dl	0.00(0.00,0.00)	0.06	--	--
Urine protein-creatinine ratio >1g/g	-0.00(-0.03,0.03)	0.89	--	--

Abbreviations: eGFR, estimated glomerular filtration rate; ECW, extracellular water; ICW, intracellular water; TBW, total body water.

## Discussion

This study assessed the relationship between body composition and physical function in patients CKD stages 1–5 not on dialysis. Patients with CKD stages 3–5 had lower handgrip strength and lower times of 30-second chair-stand than those with CKD stages 1–2. High ECW/TBW was significantly associated with low handgrip strength, and FTI was negatively correlated with times of 30-second chair-stand after adjustment for traditional risk factors for physical dysfunction in CKD patients.

To our knowledge, this study is the first to evaluate the association between fluid status and physical function in CKD population, and we found that fluid overload was significantly associated with impaired upper extremity muscle endurance. Accumulating evidence has documented that malnutrition-inflammation was correlated with fluid overload [22]. Stoenoiu et al. demonstrated that Inflammation restrains water channel aquaporin-1, which modulates transcellular water permeability [23]. Niebauer et al. also reported that bowel wall edema may cause increased gut permeability in patients with congestive heart failure with and fluid overload [24]. Whether malnutrition-inflammation is a cause or consequence of fluid overload is difficult to evaluate and its interaction may affect handgrip strength in CKD patients. Hypoalbuminemia has been the principal marker of malnutrition-inflammation. We stratified study participants by serum albumin cut at 4.0g/dl to analysis the effect of the interaction between malnutrition-inflammation and fluid overload on handgrip strength, and high fluid status was still associated with poor upper extremity muscle endurance (β = -91.42, P = 0.001 in high serum albumin group; β = -72.43, P = 0.01 in low serum albumin group). Therefore, abnormal fluid status is a potential predictor of upper extremity muscle endurance in CKD patients independent of malnutrition- inflammation.

Muscle structure and function are linked to physical function. A loss of lean tissue and an increase in whole-body or regional fat deposits affect muscle structure and function in older individuals [25]. In patients on maintenance dialysis, higher fat tissue and lower muscle mass were associated with higher odds of weakness and slow gait speed components of frailty [26,27]. Our present study found a strong correlation between high fat tissue index and poor lower extremity muscle endurance in CKD patients not on dialysis after adjustment for traditional predictor including BMI. The BMI and obesity have been traditionally utilized as the tool to evaluate physical function. However, decreased physical function is also found in non-obese individuals, and BMI cannot accurately investigate body composition alterations in individuals who experience a loss of lean tissue or an increase in fat tissue, even when their BMI value is within normal range or below the obese level [28].

Fat tissue is an important endocrine organ that produces pro-inflammatory cytokines, such as interleukin-6, and anti-inflammatory cytokines, such as adiponectin [28]. Excess of fat distribution may induce a loss of lean tissue through inflammatory response, which has catabolic effects on lean tissue [29]. Accumulating evidence shows that high fat mass has more adverse influence on physical function decline than low lean mass [25,30]. Marcus et al. also demonstrated that fat tissue modulates the relationship between sarcopenia and physical function [31]. In our CKD cohort, fat tissue index is positively and significantly associated with poor performance of 30-second chair stand. Nevertheless, the influence of the distribution of fat deposits on lower extremity muscle endurance is not well-known. Further studies are necessary to investigate the pathophysiologic role of fat tissue and its distribution in impaired lower extremity muscle endurance.

Hiraki et al. conducted the study to evaluate the indicators of physical dysfunction in CKD patients not on dialysis, and found that several traditional factors including aging, female, obesity and impaired renal function were associated with poor physical function [13]. Additionally, aging, sex, BMI and renal function also affect variation of body composition concurrently. In order to investigate the influence of the complicated interaction between these factors and body composition on physical function, we adjusted these traditional factors in multivariate analysis. Besides, we also stratified these CKD patients based on age of 65 year-old, sex, eGFR of 60ml/min/1.73m^2^ and the results show a significant association of handgrip strength with fluid status and LTI independent of age, sex and eGFR (S1 and S2 Figs). However, the consistent relationship between FTI and handgrip strength was found only in either CKD patients with age above 65 years or patients with CKD stages 3–5, not in younger patients and early CKD patients (S3 Fig). Relatively small numbers of subgroup patients might be one of the reasons. Nevertheless, our findings suggested a potential viewpoint of the link between body composition and physical function in CKD cohort. Clinical physicians need to closely monitor body composition in CKD patients, and this could assist in evaluating and maintaining physical function.

There was no significant correlation between abnormal body composition and cardiorespiratory endurance in our study. It is possible that body composition may have an influence on muscle endurance, instead of cardiorespiratory endurance. Our results found that baseline exercise capacity was associated with cardiorespiratory endurance. CKD patients with regular exercise habit may preserve better cardiorespiratory endurance. Whether regular intense exercise is helpful to improve physical function in CKD care is interesting to examine.

Some limitations should be considered in this study. First, this observational study described a causal relationship between body composition and physical function in CKD patients. Body composition and physical function were measured only at enrollment. The effect of time-varying body composition on physical function could not be estimated. Second, we excluded some participants, who received shunt creation and/or were unable to independently ambulate. These results could not be generalized in CKD patients not on dialysis. Third, exercise capacity was identified only by the interview. We could not evaluate the accurate amount of physical activity and exercise intensity. Finally, CKD patients usually eat a low-protein diet, and protein intake is another affecting factor of muscle strength. This study did not evaluate dietary intake in these participants. Further study is needed to measure the protein intake by nutritionists to investigate the relationship between protein intake and physical function.

## Conclusions

Our results demonstrated that high fluid status and high fat tissue index were associated with impaired upper and lower extremity muscle endurance in CKD patients. The measurement of body composition is helpful for evaluation of physical function. The underlying pathophysiological mechanisms of physical function in the changes of body composition warrant further investigation. Furthermore, the evaluation of the effect of early intervention on preserving physical function will be necessary in patients with CKD.

## Supporting Information

S1 Fig Association between fluid status and handgrip strength in patients with CKD stratified by age, sex and renal function. (TIF)

S2 Fig Association between lean tissue index and handgrip strength in patients with CKD stratified by age, sex and renal function. (TIF)

S3 Fig Association between fat tissue index and handgrip strength in patients with CKD stratified by age, sex and renal function. (TIF)
