# Supplementary figures and images for: Association of Fluid Status and Body Composition with Physical Function in Patients with Chronic Kidney Disease
Source: PLoS One. 2016 Oct 31;11(10):e0165400. doi: 10.1371/journal.pone.0165400 (PMC5087878; doi:10.1371/journal.pone.0165400)

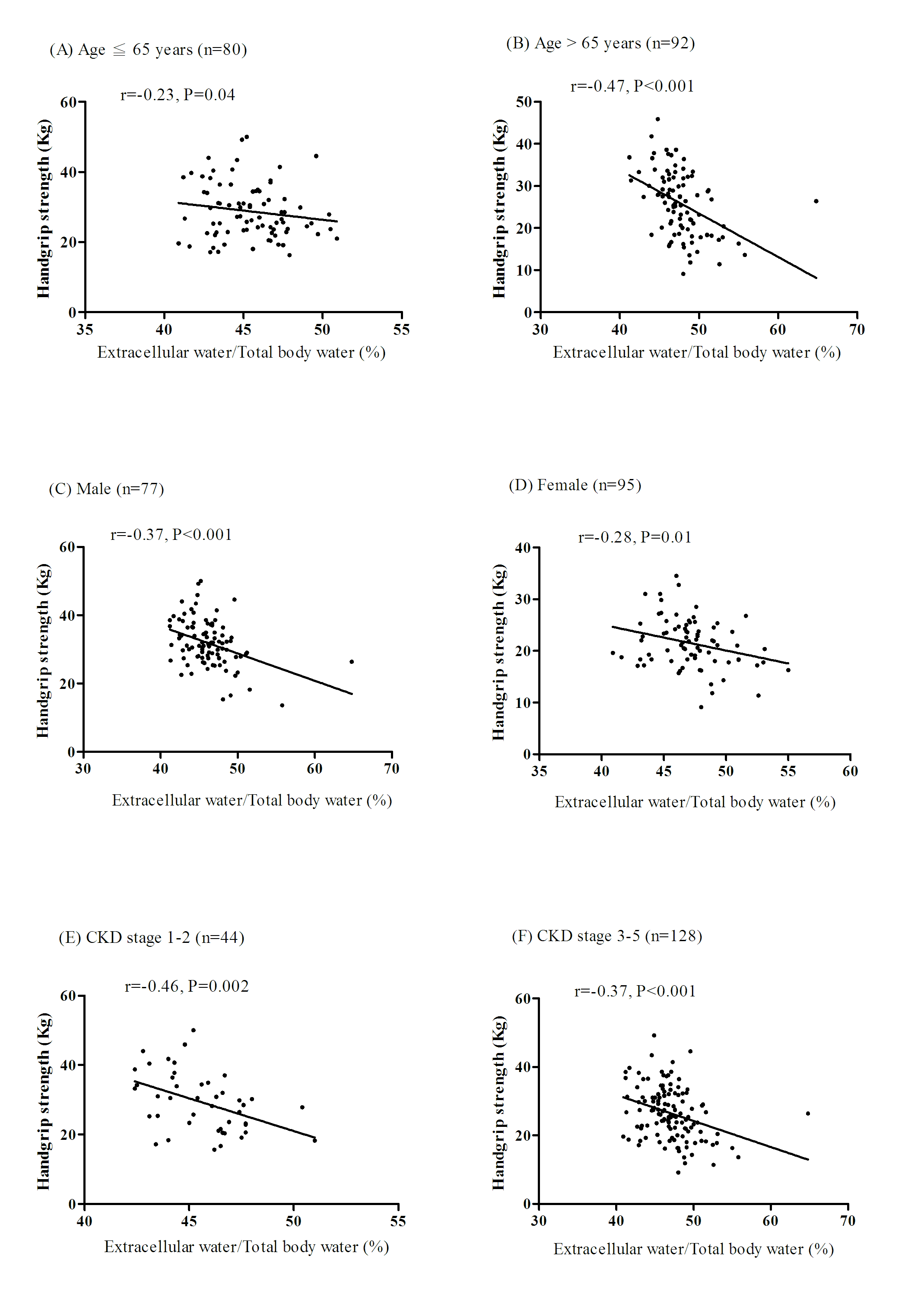

Supplement: S1 Fig — (TIF) [file pone.0165400.s001.tif]

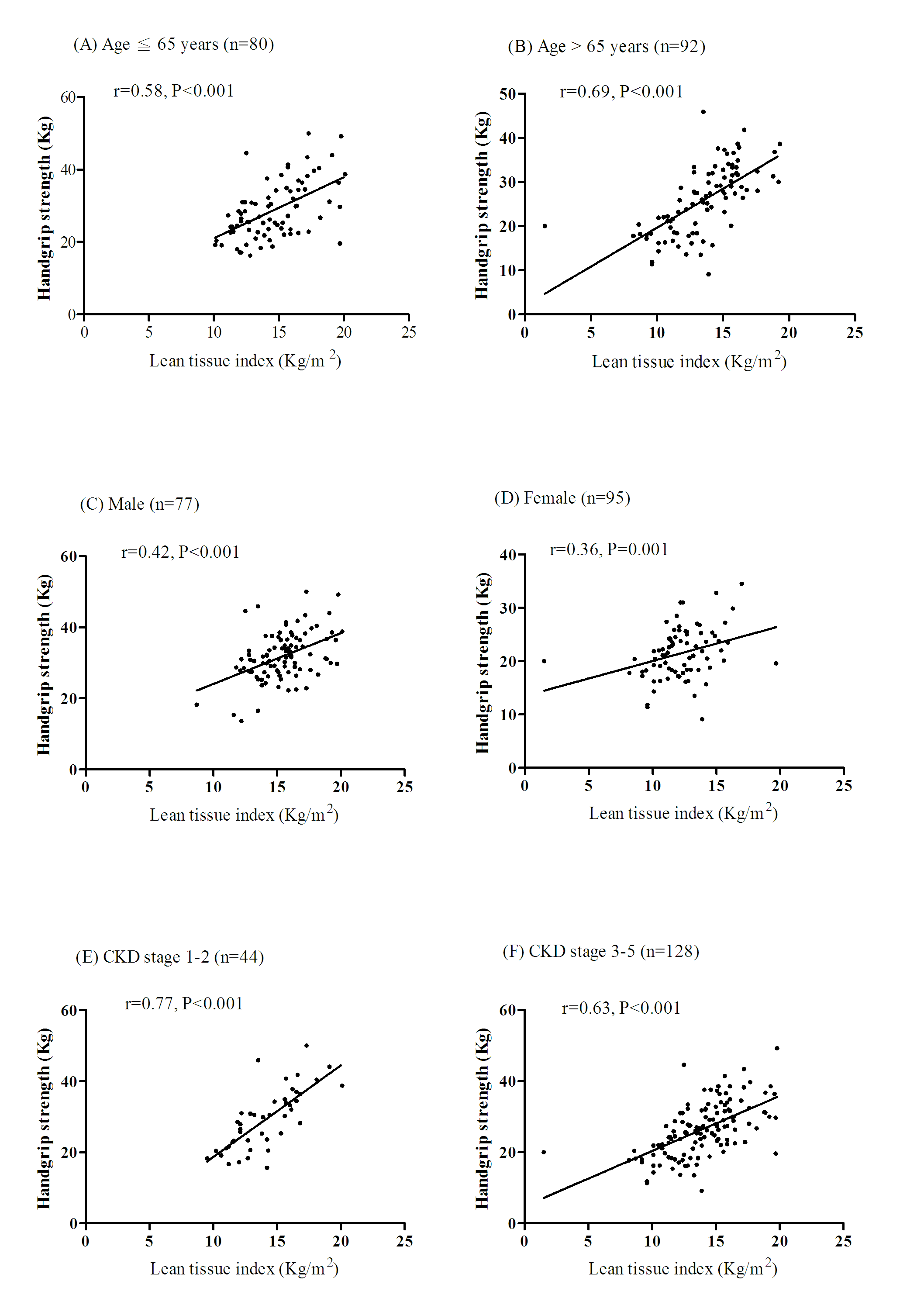

Supplement: S2 Fig — (TIF) [file pone.0165400.s002.tif]

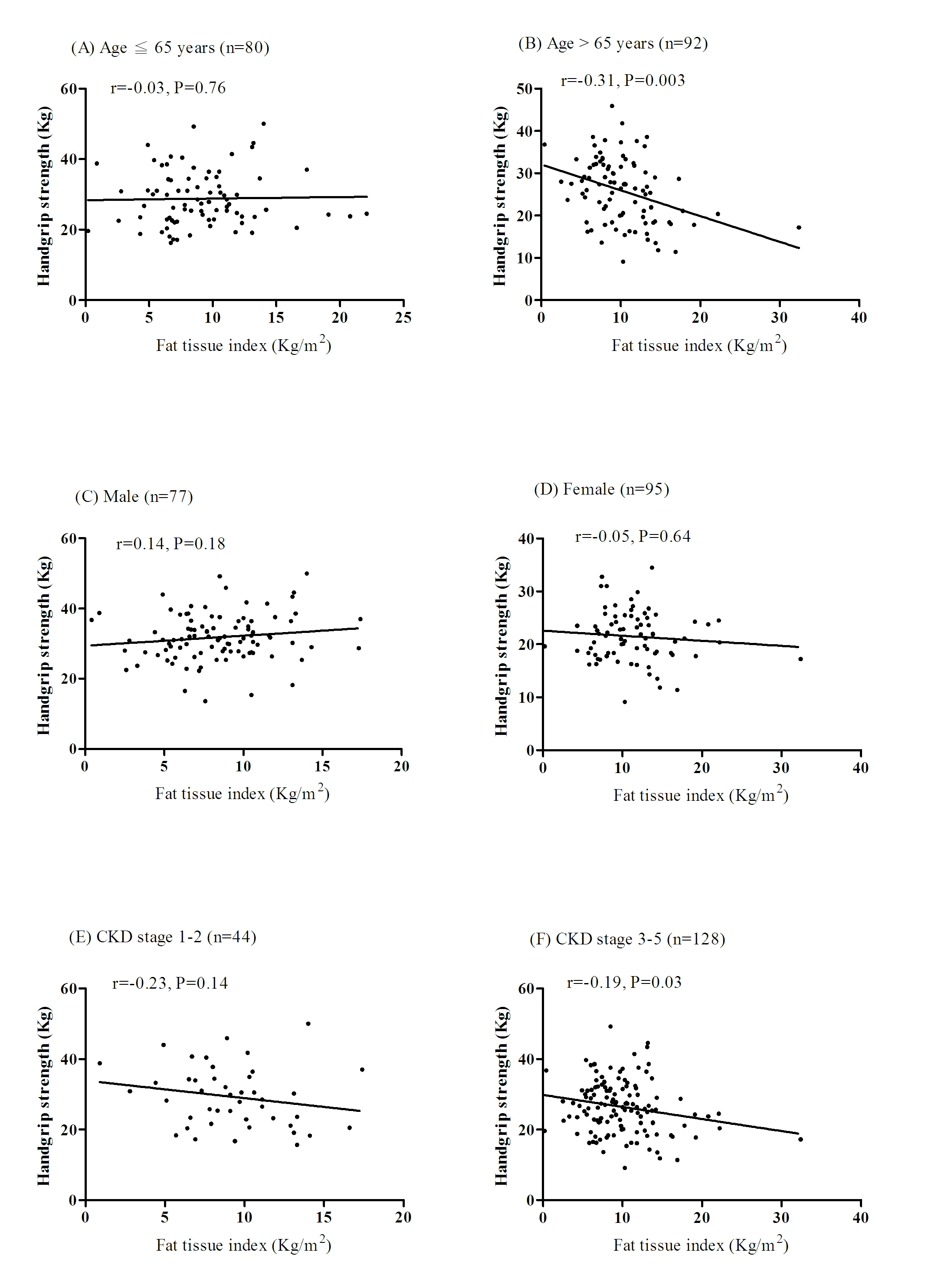

Supplement: S3 Fig — (TIF) [file pone.0165400.s003.tif]
